# Hunting practices in southwestern Amazonia: a comparative study of techniques, modalities, and baits among urban and rural hunters

**DOI:** 10.1186/s13002-023-00599-z

**Published:** 2023-07-03

**Authors:** Marcela Alvares Oliveira, Franciany Braga-Pereira, Hani Rocha El Bizri, Thais Queiroz Morcatty, Carolina Rodrigues da Costa Doria, Mariluce Rezende Messias

**Affiliations:** 1 Post-graduate Program in Biodiversity and Biotechnology of Legal Amazon (BIONORTE Network), Federal University of Rondônia, Porto Velho, Brazil; 2 Post-graduate Program in Conservation and Use of Natural Resources, Federal University of Rondônia, Porto Velho, Brazil; 3 Research Network on Diversity, Conservation and Use of Amazonian Fauna (RedeFauna), Manaus, Brazil; 4 ComFauna, Comunidad de Manejo de Fauna Silvestre en la Amazonía y en Latinoamérica, Iquitos, Peru; 5 Department of Ecology and Systematics, Federal University of Paraíba, João Pessoa, Brazil; 6 School of Science, Engineering and Environment, University of Salford, Salford, UK; 7 Terrestrial Vertebrate Ecology Research Group, Mamirauá Sustainable Development Institute, Estrada do Bexiga, Tefé, Brazil; 8 Oxford Wildlife Trade Research Group, Faculty of Humanities and Social Sciences, Oxford Brookes University, Oxford, UK

**Keywords:** Hunting strategies, Wildlife use, Harvest, Lures, Amazon

## Abstract

**Background:**

Hunting is a vital means of obtaining animal in various human populations. Hunters rely on their knowledge of species ecology and behavior to develop and employ hunting techniques and increase their chances of success. The comparison of the hunting practices of different human societies can shed light on the sustainability of hunting and the impact it has on species’ populations. In this study, we examine and compare the techniques, modalities, and baits used by urban and rural hunters in Rondônia, a state in southwestern Amazonia, Brazil. We expected that rural hunters would use these elements and have greater knowledge when compared to urban hunters. We also expect that the use of specific hunting techniques and modalities will have greater selectivity and specificity of capture for rural hunters and that this knowledge will differ between groups.

**Methods:**

We conducted 106 semi-structured interviews with rural and urban hunters from October 2018 to February 2020. We analyzed the data using PERMANOVA and Network analyses to compare and contrast the hunting practices of each group.

**Results:**

We recorded four main hunting techniques divided into ten modalities with three techniques and seven modalities being the preferred choices among hunters. Waiting for at a Fruit Tree was cited as the primary technique employed by hunters living in urban and rural areas indicated. While the techniques and modalities were similar among hunters, the composition of species targeted and baits used differed between groups. Our network approach showed that modularity in urban areas was numerically lower than in rural areas. All species had one to more techniques associated with their capture.

**Conclusions:**

Hunters living in urban and rural environments showed high similarity in their practices, probably due to sharing similar environments to hunt containing similar species, as well as targeting preferably the same species.

**Supplementary Information:**

The online version contains supplementary material available at 10.1186/s13002-023-00599-z.

## Background

Wildlife is an important source of animal protein, essential to the diet of different human populations [1]. Hunting is the usual means to harvest wildlife, and the success of capture of the techniques use to play an important role in daily protein intake. More isolated populations in tropical forests Amazon have restricted access to industrial or commercial sources of protein. In addition, these populations also face limited availability of electricity, which makes it even more difficult to store meat for long periods. These conditions impose the need for a greater frequency of hunting [2, 3]. Hunting practices are a complex process that involves knowledge inherited from initiation figures and adapting to variations in the availability of target species [4]. It may involve the use of lures in different hunting and attraction contexts [5, 6], types of environments [7, 8], and different techniques [9, 10]. This includes both active and passive hunting techniques [6, 11], which can be performed individually or in groups, depending on the species intended to be captured [11–15]. All these factors are taken into consideration by hunters in decision-making regarding the hunting strategy to be employed [16].

A hunting strategy can be defined as the set of factors that are analyzed for decision-making regarding hunting, which include departure time, capture location, and technique to be employed. The hunting technique, on the other hand, consists of the method used to capture the target species [9, 10, 17]. The hunting modality comprises the variations in the format or procedure of a given technique [10, 17, 18].

Understanding the specific hunting techniques and modalities used is crucial for effective management and conservation of hunted species. These studies are, however, scarce. In the Amazon rainforest, while there have been several studies in the Amazon that have examined hunter profile and hunting/consumption patterns, food taboos, and food preference [19–23], there has been a limited focus on the specific hunting techniques and modalities employed by people living in this biome. These few studies have shown that the capture of yellow-footed tortoises (*Chelonoidis denticulatus*) involves the use of different hunting techniques that are combined with knowledge of environmental characteristics to maximize the harvest of individuals [6, 11, 24]. The hunting of armadillos (genus *Dasypus*), on the other hand, comprises the combination of two techniques: specialized dogs and jiquis—cylindrical wire traps inserted into the opening of burrows [18].

Traditional hunting practices can generate information that can be incorporated into scientific capture methods [24], biodiversity monitoring programs [25–27], and management of hunted species [27, 28]. In the case of lowland pacas (*Cuniculus paca*), hunting methods such as chasing with dogs, used frequently by Amazonian local communities, generate a higher capture rate when compared to conventional scientific methods such as live trapping, besides having a lower cost [25]. Hunting activity is not restricted to rural residents in the Amazon, although studies are focused on riverine, indigenous, extractivist or settled populations [29–32]. Residents of urban centers frequent the rural or peri-urban environment to hunt for either food supplementation or commercialization [20]. Comparative analysis between these two types of hunters in the Amazon is restricted to the use of zootherapeutics [33] and the relationship of potentially hunted species with those captured [34]. In general, these hunters conduct hunting to meet different motivations, which include commercialization, medicinal use, subsistence, conflict retaliation, and recreation [20, 28, 35–37]. However, for the state of Rondônia, in-depth scientific studies on hunting techniques are incipient, presenting only the frequency of use of techniques [38] without comparing different groups of hunters. Ramos et al. [38] studying hunters from a village of waste pickers found that waiting at a fruit tree and foot tracking were the preferred techniques of 64.29% and 45.86% of the interviewees.

To determine whether urban hunters differ from rural hunters, we analyzed the different techniques, and preferences for hunting techniques employed. As hunters use elements of the natural environment or introduced hunting techniques to attract animals, such as the use of fruits, to maximize hunting success, we expected that rural hunters would use these elements and have greater knowledge when compared to urban hunters due to their greater connection with the rural environment. We also expect that the use of specific hunting techniques and modalities will have greater selectivity and specificity of capture for rural hunters and that this knowledge will differ between groups.

## Methods

### Study area

**Fig. 1:**
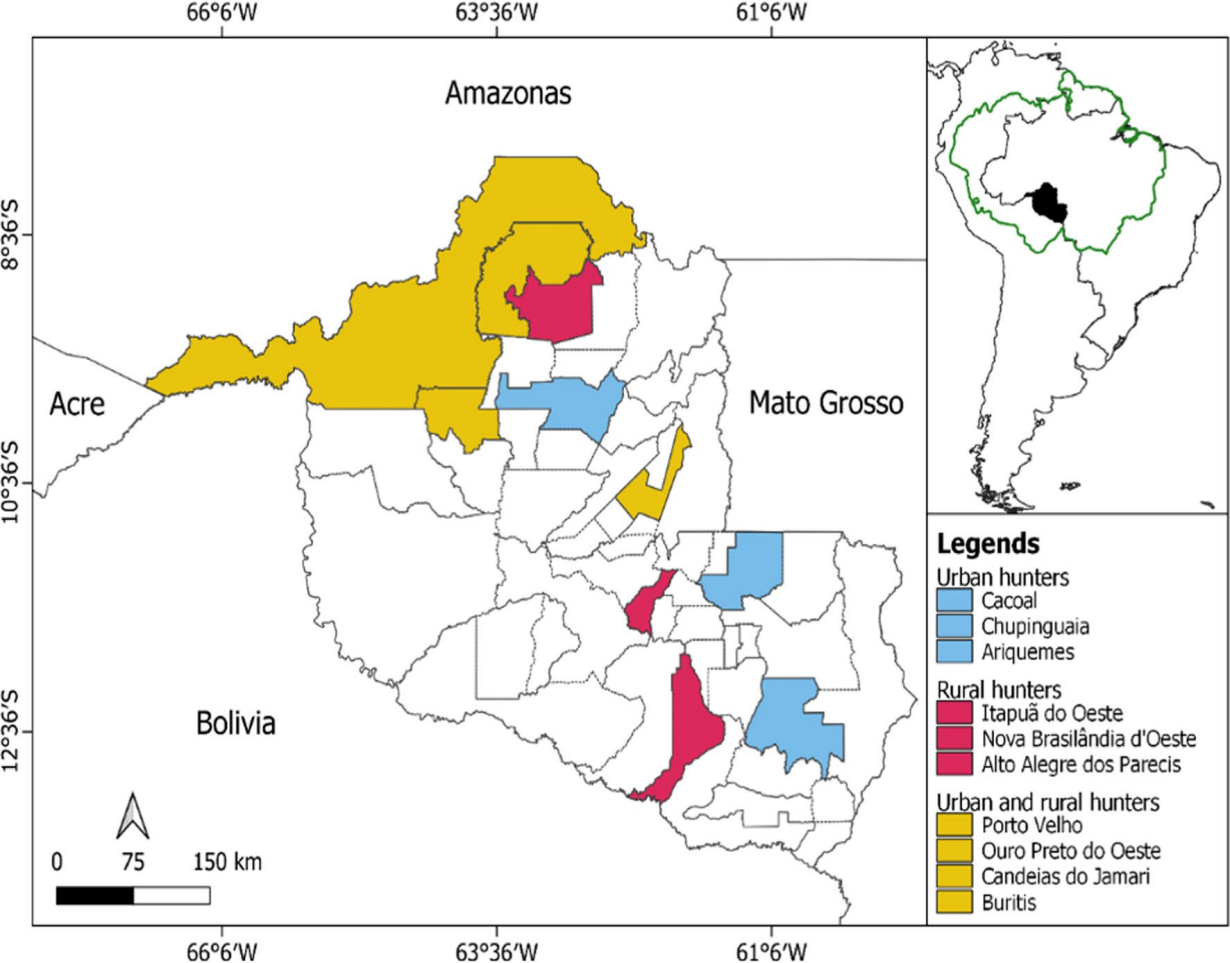
Municipalities in the state of Rondônia in which the interviewed hunters reside

The study was conducted in the state of Rondônia, Brazil, which is located in the southwestern Amazon, having an area of approximately 237,000 km^2^ and 1,777,225 inhabitants, with a predominance of an urban population (1,149,180 inhabitants) [39]. The predominant climate in Rondônia is humid continental equatorial, characterized by an average annual rainfall between 2000 and 2300 mm. It has high temperatures throughout the year (24–27 °C), with a dry season between June and August, with maximum temperatures reaching 37 °C [40]. The hydrography is integrated into the Amazon Basin, formed mainly by the Madeira River [41]. The state comprises three important biomes: Amazon Rainforest, Pantanal, and Cerrado. The Open Ombrophilous Forest is the predominant forest typology in the state, as well as in the studied municipalities, covering 55% of the total vegetation area [42]. The state has 52 municipalities, and interviews were conducted in 10 of them (Fig. 1). Between October 2018 and February 2020, we interviewed 106 hunters, 49 urban and 57 rural.

### Data collection

Data were collected using semi-structured questionnaires [43]. The choice of participants was made through the Snowball method [44], which consists of selecting people recognized by the community as experts on the subject investigated, in this case, wild animals and hunting activity. The categorization of hunters into rural and urban followed the proposed by Oliveira et al*.* [34], using as criteria the place of fixed residence, length of stay in the environment of 90% per week, and self-declaration. All the self-designated urban hunters lived within the urban perimeters of the investigated cities, while the rural hunters lived beyond the peri-urban perimeter or the urban expansion zone of the cities, totally inserted in the rural environment. All hunters interviewed were over 18 years old and residents of Rondônia for at least six months. All hunters were asked the following questions during the interviews: place of residence, hunting techniques used, preference for hunting techniques, species-specific hunting techniques, and description of the last five hunting events (species, techniques, and time).

All hunters were informed about the objectives of the project and guaranteed that their names would not be disclosed, as determined by Resolution CNS 466/12 on research involving human beings. This study was approved by the Research Ethics Committee (CEP) of the Aparício Carvalho University Center under protocol number 2.661.332.

### Data compilation

For this paper, we categorized as techniques the main mode of capture and as modality the variations related to the main technique. Regarding autonomy, the techniques were categorized as active or passive, as proposed by Fernandes-Ferreira [17]. Active techniques encompass those in which hunters actively seek the target species and are present at the moment of capture, using firearms, bladed weapons, or manual capture. Passive techniques involved the use of traps that do not require the presence of the hunter to capture the species.

### Statistical analysis

We performed a Permutational Multivariate Analysis of Variance (PERMANOVA) with 9999 permutations to test each cluster (species composition, techniques, modalities, and baits used) between rural and urban hunters. For this test, we used R software (ver. 3.5.3) [45], employing the *vegan* package [46].

To describe the hunting techniques used in Rondônia by hunters, we first used descriptive statistics. We then performed a bimodal network analysis to see the relationship between technique and species in each area, where a set of nodes represents hunting techniques, connected to the set of nodes representing the species hunted. In bimodal networks, interactions occur between different types of nodes, but not between nodes of the same type. In doing so, we show the interactions between the frequency of technique use by species topologically for each area. In addition, we also calculate one quantitative metric for each area—(a) modularity—, and three qualitative (binary) metrics: (b) average degree, (c) connectedness, and (d) nesting [47].

We tested Modularity (M) to see how well each species-specific hunting technique quantifies the inclination of nodes to cluster into cohesive groups that are more connected to each other than to other parts of the network. We analyzed Connectedness (C) to see when there was a larger number of techniques being used to catch a larger number of species, which represents the relativized number of interactions observed by all possible interactions [47]. Subsequently, Nesting (N) was analyzed to verify if certain species are captured only by some specific techniques and to measure if and when some technique is used in a more generalist way (for capturing a greater number of species) [48].

Newman's metric [49] was employed to define M by comparing its empirical value to a reference distribution of M values based on a set of 1000 theoretical matrices created by a null model in which the degrees of the species hunting technique (links) vary between zero and the average degree of the empirical network. Significance (*p* ≤ 0.05) was based on the location of the observed M relative to the 95% confidence interval derived from the null model [50]. We used the NODF metric for N, which ranges from zero, when the matrix is perfectly un-nested, to 100, when the matrix is perfectly nested [48]. We also compared the NODF value of the empirical network with a reference distribution created by 1,000 theoretical matrices generated by a null model based on a probability matrix (null model 2 of BASCOMPTE et al*.* [50]) and adopted the same criterion mentioned above for M significance. The network analyses were performed in R (ver. 3.5.3) [45] based on the bipartite package [51].

## Results

### Characterization of hunters and hunting practices

Urban hunters had a mean age of 34 ± 12 years, and 86.68% of them worked in the city. The rural hunters were 37 ± 15 years old, with 92.98% working in agricultural production. The rural hunters did not belong to any traditional community in the state (e.g., quilombolas or indigenous people), being rural producers of different sizes, living predominantly in rural settlements and isolated houses. We recorded four main hunting techniques divided into ten distinct modalities, accounting for a total of 126 technique mentions by urban hunters and 115 by rural hunters, with only one technique and modality exclusive to rural hunters, i.e., the salt bait tracking (Table 1).

**Table 1:** Description of hunting techniques and modalities mentioned and preferred among urban and rural hunters in Rondônia, Brazil

Autonomy	Technique	Mode	Description	Urban mention	Rural mention	Urban preference	Rural preference
Passive	Trap	Firearm	Use of firearms associated with a shooting trap. The traps are installed on trails of the target species, where on one side the gun is placed and a line is stretched across the trail. When the animal passes on the trail and pushes the line, it triggers the gun. The height of the gun is installed according to the height of the target species	5	1	–	–
Active	Wait	Fruit tree	The hunter waits armed (rifle or shotgun) at fruit trees used by the animals. He may choose to tie a net at a height of more than two meters, sit on a structure called a climber, or sit at ground level at a certain distance from the fruit tree	37	34	25	26
Active		Salt bait	The hunter uses containers with of cooking salt that are hung at strategic locations, which can be on the species' trails or associated with ditches, watering holes, or fruit trees, where salt drips slowly due to the humidity. The hunter waits in the area armed (rifle or shotgun) similarly to waiting by a fruit bowl	17	8	8	1
Active		Blind	The hunter prepares fruit suppers at certain locations and waits armed (rifle or shotgun) at the supper site similarly to waiting by a fruit tree	16	10	7	2
Active		Mineral lick	Installation of waiting areas in natural mudflats or sloughs that are used mainly by large species. The hunter can wait in place armed (rifle or shotgun) similarly to waiting by a fruit tree	6	6	2	–
Active	Tracking	Foot	The hunter searches armed (rifle or shotgun) for prey by walking along trails established according to the territories of the species, randomly or in ditches	22	31	8	20
Active		Canoe	Predominantly nocturnal, where hunters travel by canoe, armed (shotgun, rifle, machete, or harpoon) with flashlights or not, along the banks of bodies of water, slowly searching for the target species	14	5	1	1
Active		Dog	It differs from tracking on foot in that it employs dogs for tracking game. Hunters may or may not use rifles, shotguns, or machetes. In the case of not using guns in general, the capture is carried out by dogs	10	15	2	7
Active		Horseback	Used in pastures or crops, horses are employed to facilitate the location of prey. The hunters ride armed with rifles or shotguns	1	2	–	–
Active		Salt bait	It differs from waiting by a salt bait in that the hunter surveys the points armed (rifle or shotgun) without waiting on the spot. For this reason, several salt bait points are installed on the hunting trail system used	–	1	–	–

There was a preference for three techniques and seven modalities. Only one urban hunter reported no preference for hunting techniques.

As for the use of traps with firearms, homemade or old weapons are prioritized. These weapons, popularly called trabuco by both groups of hunters, have only the barrel and the shooting system. Six urban trappers informed that they know other trappers using *leghold traps*. This type of trap is not sold in the state but can be ordered from a locksmith shop. The same hunters informed that they were against the use of leghold traps due to the risk of accidents, the capture of non-game or juvenile animals, and the torture of the shot animals.

Another trap model reported by two rural hunters is the pigsty, also employed by other hunters. According to these hunters, this model is used mainly for capturing feral pigs, known locally as elongated pigs. It consists of using reinforced screens placed side by side in the shape of a small pigsty. A guillotine-style door is installed at the entrance, which can be operated manually or automatically with a pressure trigger system similar to that used in *Sherman* traps used to capture small mammals. This space is covered with corn, cassava, or food leftovers in general to attract the animals, and, initially, the entrance remains with the door system not activated so that the animals can get used to the trap. After the habituation period, the automatic trigger system is activated, or the trapper is positioned next to the door and activates it at his convenience.

**Fig. 2:**
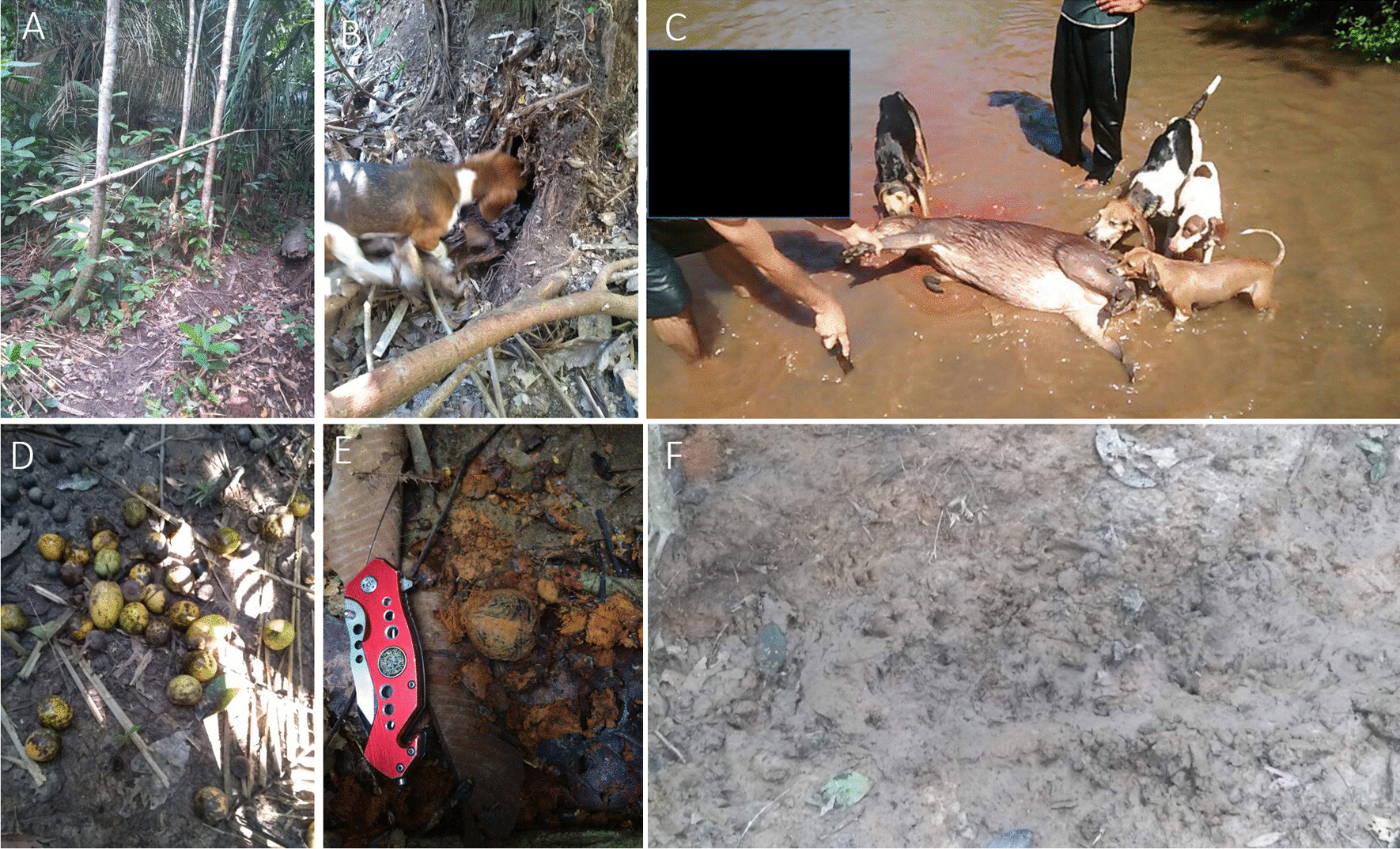
Strategies and baits used to capture wild animals in Rondônia, Brazil. **A** Waiting at a fruit tree, **B** tracking using a dog to capture paca, **C** capture of capybara using dogs, **D** mango bait, **E** tucumã bait, **F** mineral lick with recent visit of white-lipped peccary

Regarding species to be captured with specific techniques, 73.47% of urban hunters said they make use of specific techniques, however, among rural hunters, the number of affirmative answers was 59.65%. Sixteen species are captured with specific techniques by urban hunters, while ten are seized by rural hunters. Hunters living in urban and rural environments indicated waiting (84.24% and 82.57%) by fruit tree (59.78% and 55.78%) as the main technique and modality, respectively, employed to capture animals. Regarding the use of baits, 37 botanical species were mentioned, where 70.97% were native species. Paca was the species with the largest number of citations of specific techniques and number of baits used by both urban and rural hunters. The techniques (PERMANOVA *F* = 0.348; *p* = 0.7) and modalities (PERMANOVA *F* = 0.0983; *p* = 0.8) employed were similar between urban and rural hunters. The composition of species (PERMANOVA F = 3.82; *p* = 0*.*01) and baits mentioned (PERMANOVA *F* = 5.61; *p* = *0.*01) differed significantly, with urban hunters reporting a larger number of species and baits (Table 2 and Fig. 2).

**Table 2:** Species and specific techniques, modalities, and baits employed by hunters in the state of Rondônia, Brazil

Technique	Mode	Bait	Scientific Name	Prey	Urban	Rural
Wait	Fruit tree	Babaçu	*Attalea speciosa*	*Tapirus terrestris*	1	0
				*Mazama americana*	1	0
				*Tayassu pecari*	1	0
				*Dicotyles tajacu*	1	0
				*Cuniculus paca*	2	0
				*Dasyprocta* spp	1	0
		Baginha	*Stryphnodendron pulcherrimum*	*Tapirus terrestris*	1	0
				*Mazama americana*	2	0
				*Tayassu pecari*	1	0
				*Cuniculus paca*	9	0
		Banana	Musa paradisiaca*	*Cuniculus paca*	1	0
		Brejauba	*Astrocaryum aculeatissimum*	*Dicotyles tajacu*	0	1
				*Cuniculus paca*	0	1
		Buriti	*Mauritia flexuosa*	*Dasypus beniensis*	1	0
				*Priodontes maximus*	1	0
				*Dasypus novemcinctus*	1	0
				Dasypodidae/Chlamyphoridae	1	0
				*Tapirus terrestris*	1	0
				*Mazama nemorivaga*	1	0
				*Dicotyles tajacu*	1	1
				*Cuniculus paca*	3	2
		Camaru	*Dipteryx odorata*	*Mazama americana*	1	0
		Caucho	*Castilla ulei*	*Dasypus beniensis*	0	1
				*Mazama americana*	1	1
				*Dicotyles tajacu*	0	1
				*Cuniculus paca*	0	1
		Copaiba	*Copaifera langsdorffii*	*Dasypus beniensis*	1	0
				*Priodontes maximus*	1	0
				*Dasypus novemcinctus*	1	0
				*Tapirus terrestris*	1	0
				*Mazama americana*	1	0
				*Mazama nemorivaga*	1	0
				*Tayassu pecari*	1	0
				Tinamidae	1	0
				*Pauxi tuberosa*	1	0
		Embira	*Xylopia* sp	*Dasypus beniensis*	0	1
				*Mazama americana*	0	1
				*Cuniculus paca*	1	1
		Bean	*Parkia* sp	*Tapirus terrestris*	1	0
				*Mazama americana*	1	0
				*Tayassu pecari*	1	0
		Figueira	*Ficus* sp	*Cuniculus paca*	1	0
		Goiaba do mato	*Bellucia grossularioides*	*Tapirus terrestris*	0	1
				*Cuniculus paca*	1	1
		Jambo-do-mato	*Syzygium* sp	*Mazama americana*	2	0
				*Cuniculus paca*	1	0
		Red Jambo-Red	Syzygium malaccense*	*Dasypus novemcinctus*	1	0
		Jatoba	*Hymenaea courbaril*	*Tapirus terrestris*	1	0
				*Mazama americana*	1	0
				*Tayassu pecari*	1	0
		Jenipapo	*Genipa americana*	*Mazama americana*	1	0
				*Cuniculus paca*	1	0
		Papaya	Carica papaya*	Cuniculus paca	0	1
		Mango	Mangifera indica*	*Tapirus terrestris*	0	1
				*Cuniculus paca*	0	1
		Passion Fruit	*Passionflower* sp	*Cuniculus paca*	1	0
		Mirindiba	*Lafoensia glyptocarpa*	*Dasypus novemcinctus*	0	1
				*Tapirus terrestris*	0	1
		Oreinha	*Enterolobium contortisiliquum*	*Mazama americana*	1	0
		Ouricuri	*Syagrus coronata*	Dasypodidae/Chlamyphoridae	1	0
				*Mazama nemorivaga*	1	0
				*Dicotyles tajacu*	1	0
				*Cuniculus paca*	1	0
		Piquiá	*Caryocar villosum*	Dasypodidae/Chlamyphoridae	1	0
				*Mazama nemorivaga*	1	0
				*Tayassu pecari*	0	1
				*Dicotyles tajacu*	1	0
				*Cuniculus paca*	4	1
		Pupunha	*Bactris gasipaes*	*Cuniculus paca*	1	0
				*Dasyprocta* spp	1	0
		Tucumã	*Astrocaryum aculeatum*	*Dasypus beniensis*	2	1
				*Priodontes maximus*	1	0
				*Dasypus novemcinctus*	1	1
				Dasypodidae/Chlamyphoridae	2	0
				*Tapirus terrestris*	1	2
				*Mazama americana*	1	0
				*Mazama nemorivaga*	1	0
				*Tayassu pecari*	1	1
				*Dicotyles tajacu*	1	1
				*Cuniculus paca*	13	17
				*Dasyprocta* spp	2	0
		Uxi	*Endopleura uchi*	Dasypodidae/Chlamyphoridae	1	0
				*Mazama nemorivaga*	1	0
				*Tayassu pecari*	0	1
				*Dicotyles tajacu*	1	0
				*Cuniculus paca*	1	3
Wait	Fruit bait	Babaçu	*Attalea speciosa*	*Cuniculus paca*	0	2
		Baginha	*Stryphnodendron pulcherrimum*	*Cuniculus paca*	2	6
		Apple	Malus domestica*	*Cuniculus paca*	1	0
		Cassava	*Manihot esculenta*	*Cuniculus paca*	1	0
		Mango	Mangifera indica*	*Cuniculus paca*	5	5
				*Dasyprocta* spp.	1	0
		Corn	Zea mays*	*Cuniculus paca*	2	3
		Pupunha	*Bactris gasipaes*	*Cuniculus paca*	1	0
				*Dasyprocta* spp.	1	0
		Soy	Glycine max*	*Cuniculus paca*	0	1
		Tucumã	*Astrocaryum aculeatum*	*Dasypus beniensis*	1	2
				*Dasypus novemcinctus*	0	1
				*Cuniculus paca*	3	5
Wait	Flower	Embira	*Xylopia* sp.	*Mazama americana*	1	0
		Piquiá	*Caryocar villosum*	*Mazama americana*	1	0
		Brazil Nut	*Bertholletia excelsa*	*Cuniculus paca*	1	0
		Embira	*Xylopia* sp	*Cuniculus paca*	1	0
Wait	Salt bait	Kitchen salt	–	*Dasypus novemcinctus*	0	0
			–	*Tapirus terrestris*	0	1
			–	*Mazama americana*	1	1
			–	*Tayassu pecari*	2	3
			–	*Dicotyles tajacu*	4	4
			–	*Cuniculus paca*	3	1
			–	*Dasyprocta* spp	0	1
			–	*Pauxi tuberosa*	1	0
			–	*Penelope jacquacu*	1	0
Wait	Mineral lick	–	–	*Mazama americana*	1	1
		–	–	*Tayassu pecari*	2	0
		–	–	*Dicotyles tajacu*	4	0
		–	–	*Cuniculus paca*	3	0
		–	–	*Pauxi tuberosa*	1	0
		–	–	*Penelope jacquacu*	1	0
Tracking	Foot	Baginha	*Stryphnodendron pulcherrimum*	*Cuniculus paca*	1	0
		Copaiba	*Copaifera langsdorffii*	*Cuniculus paca*	1	0
		Guava	*Bellucia grossularioides*	*Cuniculus paca*	1	0
		Souva	Unidentified	*Cuniculus paca*	1	0
		Tucumã	*Astrocaryum aculeatum*	*Cuniculus paca*	1	0
		Uxi	*Endopleura uchi*	*Cuniculus paca*	1	0
		–	–	*Dasypus beniensis*	1	0
		–	–	*Priodontes maximus*	1	0
		–	–	*Dasypus novemcinctus*	3	0
		–	–	Dasypodidae/Chlamyphoridae	0	1
		–	–	*Tayassu pecari*	1	1
		–	–	*Cuniculus paca*	3	1
		–	–	*Hydrochoerus hydrochaeris*	2	2
		–	–	Alligatoridae	2	0
Tracking	Dog	–	–	*Dasypus novemcinctus*	1	0
		–	–	*Tapirus terrestris*	0	1
		–	–	*Tayassu pecari*	0	2
		–	–	*Dicotyles tajacu*	0	1
		–	–	*Cuniculus paca*	2	1
		–	–	*Hydrochoerus hydrochaeris*	1	1
Tracking	Canoe	–	–	*Cuniculus paca*	2	0
		–	–	Alligatoridae	1	3
Tracking	Salt bait	–	–	*Tayassu pecari*	0	1
		–	–	*Dicotyles tajacu*	0	2
Tracking	Mineral lick	–	–	*Dicotyles tajacu*	0	1
Trap	Firearm	–	–	*Dasypus novemcinctus*	1	0
		–	–	*Cuniculus paca*	1	0

*exotic

The fruits of the botanical species buriti, babaçu, and tucumã were especially highlighted by five rural and four urban hunters, who stated that there are varieties of these fruits with low palatability, being very bitter and thus rejected by the animals. For this reason, it is necessary to choose trees that produce the sweetest and most attractive fruits. During an interview with three rural hunters, it was possible to visit an area that had both bitter and sweet babaçu trees, and it was possible to observe that the bitter fruits accumulated under the trees without being consumed, unlike the sweet fruits. Regarding mangoes, four rural and three urban hunters stated that the paca consumes only the seeds, ignoring the fruit pulp. All hunters stated that the fruit trees used for waiting are previously inspected to check if the animals of interest visit them. At the time of the hunt, the trees are again inspected and those with the most recent animal activity are chosen. All of the 16 urban hunters who reported the use of copaíba informed that the animals use the fruit of this species when they have a large infestation of worms and that the fruit is consumed by the animals for medicinal purposes. This statement is based on the observation of animals with large infestations caught waiting near copaíba trees.

**Fig. 3:**
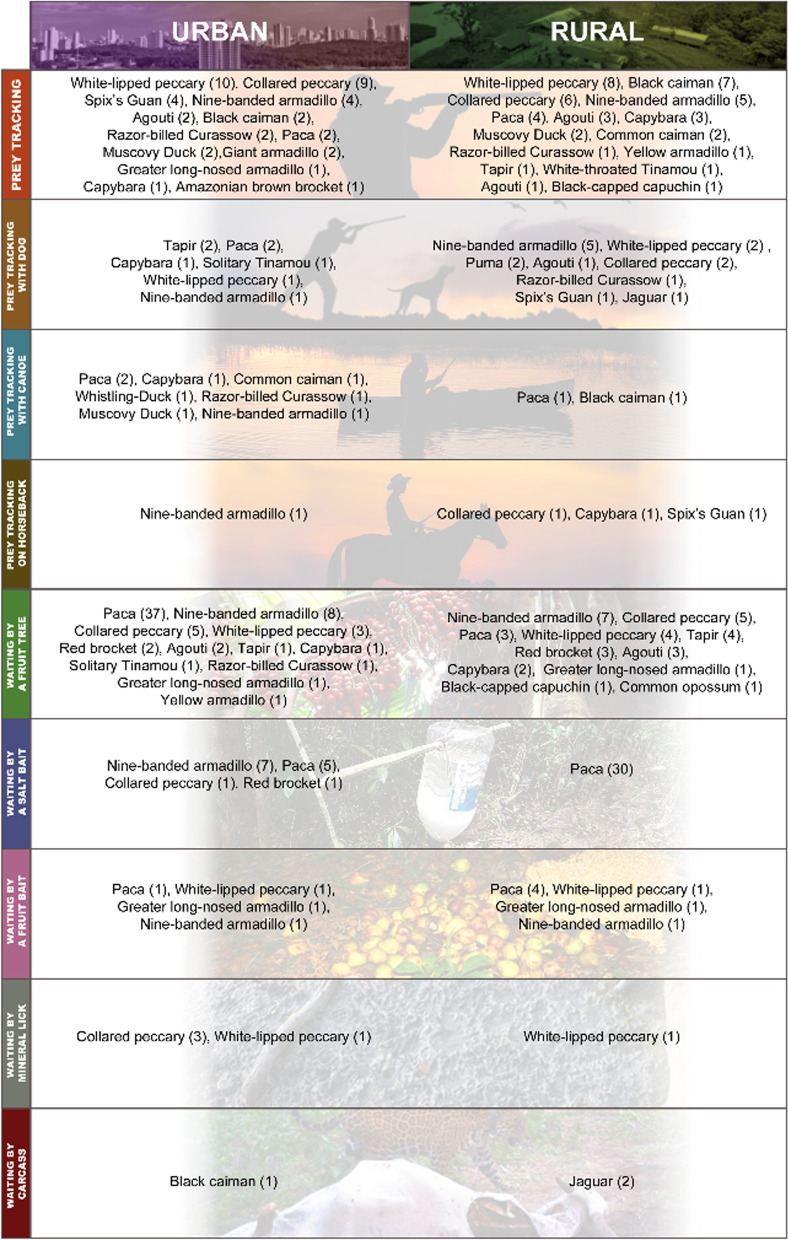
Comparison of the use of active techniques from the last hunting events

Analyzing the last five hunting events, hunters reported 306 captures, of which 160 were taken by urban hunters and 146 by rural hunters, employing two techniques and eight modalities. In these records, the use of domestic animal remains as bait to capture wild cats and caimans is cited, which were not previously mentioned. Our results indicate a greater use of active hunting techniques for the capture of paca. Analyzing the last five hunting events, urban hunters killed 48 pacas, where 87.5% of the events used the waiting technique. Rural hunters killed 46 individuals, employing passive methods in 80.43% of the events. Nighttime was the time of greatest capture, with 59.38% of urban and 62.33% of rural individuals, following the pattern of preference (*χ*^2^ = 2.91, *df* = 4, *p* = 0.56). Waiting was the most recorded technique among urban hunters (56.71%), while the recorded values of rural hunters of the use of waiting (51.37%) and tracking (48.63%) were similar, a pattern observed in technique preference (Fig. 3).

### Network topology and metrics

**Fig. 4:**
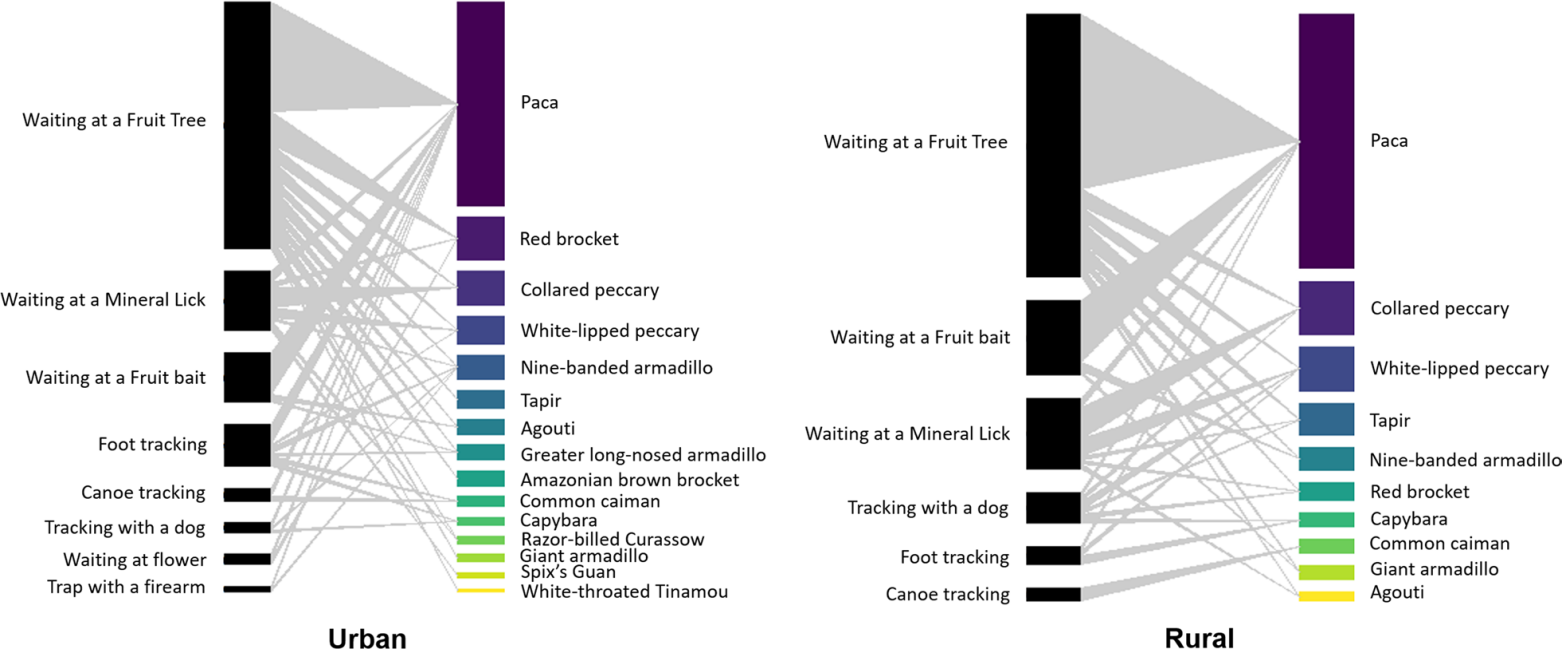
Network analysis regarding hunting strategies and target species of hunters in Rondônia, Brazil

Our network approach showed that modularity (M) was small for both areas, but in the urban area, it was numerically smaller than in the rural area [Murban = 0.24; Mrural = 0.28] (Fig. 4). Among urban hunters, each technique mentioned is used for at least two species, while among rural ones, there is one technique applied for the capture of only one species (canoe used for caiman capture). However, we found a significant difference between the empirical modularity and the respective null models for all groups analyzed [Murban_null = 0.13; *p* < 0.001; Mrural_null = 0.14; *p* < 0.001] (Additional file 1). We found lower connectedness (C) for rural compared to urban areas [Curban = 0.30; Crural = 0.45], as all species had more techniques associated with capture by hunters from rural than urban areas. We found nesting (N) for both areas [Nurban = 59.9; Nrural = 45.5]. However, this nesting did not show statistical significance when compared to that expected at random [Nurban_null = 39.8; *p* = 0.74; Nrural_null = 51.40; *p* = 0.07] (Fig. 4).

## Discussion

Our results present details on different hunting techniques, modalities, and baits used by urban and rural hunters in the Amazon, as well as a comparison between these hunting groups. Our records highlight that the use of a wide combination of techniques, modalities, and baits favors greater success and diversity of capture. Fruit trees are the most preferred among hunters, and their distribution determines the choice of hunting grounds. These locations provide a greater chance of capturing animals. For this reason, the technique of “Waiting at a Fruit Tree” is the most cited as specific by both hunter groups and is indicated for the capture of all species, except the caiman and capybara. For these animals, more active methods represent a higher probability of capture.

The number of botanical species used demonstrates a broad knowledge of the diet of game species. Furthermore, the use of exotic botanical species (soy and corn) demonstrates adaptation in the use of new resources. In rural areas, conflicts with wild animals are common due to the invasion of plantation areas and their destruction, where animals are slaughtered [52–54] and used as food [55–57]. Subsequently, the knowledge about the exploitation of these resources by animals is combined with hunting, promoting the use of these species as hunting baits.

The potential medicinal use of plants by Neotropical mammals has been recorded for different species [58–61]. In these records, it is reported that primates crush or chew the leaves and rub the chewing product on different parts of the body, especially in the regions with less hair density, such as the abdomen, not being consumed after this process. From these observations, the researchers suggest the possibility of self-medication, especially since the plants used have proven repellent or antiparasitic properties and are widely used in traditional medicine. Kaisin et al*.* [62] recorded, in the Atlantic Forest, ten species of mammals, including deer and collared peccaries, licking the sap or rubbing their bodies on the trunk of cabreúvas (*Myroxylon peruiferum*), a species with proven antiparasitic and repellent properties, hypothesizing the possibility of self-medication. Copaíba (*Copaifera langsdorffii*) oil presents multiple medicinal applications with scientific proof [63], and its potential vermifuge action may be a new line of investigation. The results presented here indicate the need for continued investigations to certify its potential consumption for self-medication.

Recording bait from hunters' knowledge is an important tool for providing data that could fill the gap in the feeding ecology of hunted species [11] and assessing the impact of hunting and exploitation of environments [23]. The tucumã, baginha, babaçu and buriti stood out as the plant species most used as baits for different game species. The tucumã has a grouped distribution [64], which can favor its use as both a food source and a bait with greater efficiency by hunters.

Rural hunters perform a series of daily work activities in the forest and in the environments they alter, which include crop/root maintenance and collection of non-timber products. During the performance of these activities, especially when moving, it is common for hunters to carry their weapons [4, 20]. Thus, if they locate a species of interest, they can capture it [65]. Oliveira and Calouro [66] state that tracking techniques require a quick response from the gunshot and, thus, the prey cannot be analyzed to assess, for example, the age or gestational period. The use of active techniques in which the hunter waits at fixed points for the target species would be more selective and sustainable. Similarly, the recording of a smaller number of species and specific baits by rural interviewees may be an effect of the knowledge of the locality, where these hunters know the efficiency of the baits and their relationship with the capture of species, focusing their capture effort on a specific group of baits.

Dogs are useful elements in hunts due to their ability to track, capture, and kill prey [12], especially fossorial and semi-fossorial animals [36, 67], although they are employed occasionally in certain localities [66]. They also represent the increased probability of encountering and capturing animals with nocturnal habits during the day [65, 68]. The use of dogs in hunting, however, divides opinions. They are mainly employed to capture mammals, [69, 70], but, in certain localities, their use is banned due to the potential of capturing young animals and scaring away fauna [20, 66, 71–74]. Constantino et al. [70], in their study with Huni Kuin indigenous hunters in Acre, demonstrated that the diversity of prey captured using dogs is lower compared to methods without dogs, in addition to focusing their capture efforts on rapidly reproducing species. Koster [75] in his study with indigenous people from the Bosawas Biosphere Reserve in Nicaragua recorded that dogs are used as a substitute for firearms to capture animals and are more efficient in locating different species, including slow-reproducing species such as tapirs. Additionally, the use of dogs increased the encounter with cats, which were consequently killed, as well as species that are not hunted/consumed.

The technique to be employed varied according to the capture target [16]. The greater record of specific techniques for the paca (*Cuniculus paca*) may be related to the higher capture rate of this species [34] in different localities in the Amazon [76]. Thus, a greater search for environments to capture this species tends to favor hunters accumulating greater knowledge about its ecology. The record of the last hunting events showed that the paca was captured mainly by employing waiting fruiting tree. The fruiting of the most cited species for paca capture (*Stryphnodendron pulcherrimum* and *Astrocaryum aculeatum*) correlates with the paca’s period of pregnancy, lactation, and weaning of their offspring, so the technique can potentially lead to killing females in the reproductive period, which is highly impactful for the maintenance of their populations and the sustainability of hunting [76].

Valsecchi et al*.* [68], while studying hunters in the Amanã Sustainable Development Reserve, recorded a greater use of active methods, in which hunters actively search for pacas at night using flashlights. This shows that the type of preferred and most effective technique can vary between localities, according to species availability and, above all, cultural influence.

The network analysis on the technique and specific capture modality showed that there is no specificity but rather a broad sharing of the use of techniques and modalities for the capture of different species. There is no relationship of specificity or selectivity for the capture of species by a particular technique and vice-versa, but rather certain techniques combined with appropriate bait and modality increase the chance of capturing different target species.

Baits (plant or otherwise) are widely employed by hunters. The main botanical baits are not species-specific, have greater spatial distribution, and some occur densely. At the same time, in the case of waiting or tracking with hunting baits, hunters distribute their baits in a way that maximizes their effort. This statement reinforces that a greater range of techniques favors a greater capture of species. The increase in bait diversity also increases the diversity of prey of potential capture. Both hunter groups do not randomly choose the trees to be used but combine constant use with traces of recent use, enhancing the chances of capture. At the same time, the passive technique combined with searching may have a greater chance of capture than active techniques, which require tracks and other traces of the animals to be located before they can be pursued and captured. At the same time, there is less energy expenditure by the hunter.

The difference in both the number of species mentioned as captured with specific techniques and the connection analysis is related to the multiple uses of the environment. Urban hunters move to areas for the sole purpose of hunting, while rural hunters involve the entire environment in their daily work activities, resulting in a higher encounter rate and distinct interactions. For this reason, rural hunters exemplify a larger number of species (1.5x) being taken with certain techniques.

## Conclusions

Our hypothesis that rural hunters have greater knowledge of hunting elements and use different techniques than urban hunters was refuted. Urban and rural hunters showed high similarity concerning techniques and modalities. The difference in reporting between species and baits may be because rural hunters have greater daily use of forest environments because of their daily practices. Such similarity can be explained by the sharing of similar environments, species distribution, and especially the great similarity in the reporting of the species captured by each technique. The appropriate choice of technique, modality, and bait produces more efficient results, and there is no species specificity of capture depending on the technique. Future studies need to focus on the efficiency of the combination of techniques, modalities, and baits for capturing species, validating the knowledge of hunters. Another focus should be given to the issue of fruit palatability and the visitation rate of game species, as well as investigating the popular knowledge of other species of potential medicinal use by the animals.

## Supplementary Information

**Additional file 1.** Details of the full models and the null model using a generalized linear model to check the relationship of hunting motivations in urban and rural huntersand personal variables in urban and rural hunters.

## Data Availability

The data used to support the findings of this study are available from the corresponding author upon reasonable request.
