# Supplementary material for: Hunting practices in southwestern Amazonia: a comparative study of techniques, modalities, and baits among urban and rural hunters
Source: J Ethnobiol Ethnomed. 2023 Jul 3;19:27. doi: 10.1186/s13002-023-00599-z (PMC10318826; doi:10.1186/s13002-023-00599-z)
Supplement: Supplementary file 1 — Additional file 1. Details of the full models and the null model using a generalized linear model to check the relationship of hunting motivations in urban and rural huntersand personal variables in urban and rural hunters. [file 13002_2023_599_MOESM1_ESM.docx]

Table S1. Details of the full models and the null model using a generalized linear model to check the relationship of hunting motivations in urban and rural hunters (model 1) and personal variables in urban and rural hunters (model 2).

| Response variable | Predictor variables | Estimate | Std. Error | z value | Pr(>\|z\|) | AIC | AIC Null model | ΔAIC |
| --- | --- | --- | --- | --- | --- | --- | --- | --- |
| model 1 | Motivation1Complementation | -1.68 | 0.74 | -2.26 | 0.02* | 173.85 | 179.26 | 5.41 |
| Locality~ main motivation for hunting | Motivation1Sports | 0.70 | 0.33 | 2.09 | 0.03* |  |  |  |
|  | Motivation1Selling | -15.50 | 1772.79 | -0.01 | 0.99 |  |  |  |
| model 2 | Income_ | 0.34 | 0.00 | 3.43 | 0.00*** | 191.40 | 195.41 | 4.01 |
| Locality~ personal | Age | -7.69 | 0.01 | -0.65 | 0.51 |  |  |  |
|  | Taught1Grandpa | -1.30 | 0.80 | -1.50 | 0.13 |  |  |  |
|  | Taught1BrotherandCousin | -0.90 | 0.70 | -1.33 | 0.19 |  |  |  |
|  | Taught1Fatherandregistration | -0.40 | 0.40 | -0.90 | 0.37 |  |  |  |
|  | Taught1Alone | -2.30 | 0.80 | -2.60 | 0.01** |  |  |  |
|  | Taught1Tio | -0.73 | 0.50 | -1.20 | 0.22 |  |  |  |
